# Large mural thrombus in the non-aneurysmal and non-atherosclerotic ascending aorta: a case report

**DOI:** 10.1186/s13019-021-01585-w

**Published:** 2021-07-23

**Authors:** Xiaoyi Dai, Chengyao Ni, Wenzong Luo, Sihan Miao, Liang Ma

**Affiliations:** 1 Department of Cardiovascular Surgery, the First Affiliated Hospital, School of Medicine, Zhejiang University, 79# Qingchun Road, Hangzhou, Zhejiang 310003 China; 2 School of Medicine, Zhejiang University, Hangzhou, Zhejiang 310003 China

**Keywords:** Thrombus, Ascending aorta, Non-atherosclerotic, Sessile, Case report

## Abstract

**Background:**

The mural thrombus in the ascending aorta is rare, most of which are associated with aneurysm or atherosclerotic lesions, with high risks of causing catastrophic thrombotic events. A mural thrombus in the non-aneurysmal and non-atherosclerotic ascending aorta is exceptionally uncommon.

**Case presentation:**

We reported a large mural thrombus in normal ascending aorta of an asymptomatic patient. Preoperative imaging confirmed the presence of the sessile thrombus located at the left anterior wall of ascending aorta. Given that it had the potential to cause fatal thrombotic complications, surgical removal and segment of ascending aorta replacement were executed. The patient had an uneventful recovery and discharged 14 days after surgery.

**Conclusions:**

Anticoagulant is the therapeutic cornerstone of ascending aortic thrombus, but surgery should be performed aggressively when the thrombus is large or floating to avoid severe embolic complications or recurrence.

## Background

The mural thrombus in the ascending aorta(AA) is rare, most of which are associated with aneurysm or atherosclerotic lesions, with high risks of causing catastrophic thrombotic events, such as ischemic stroke, acute myocardial infarction and peripheral arterial embolism. A mural thrombus in the non-aneurysmal and non-atherosclerotic AA is exceptionally uncommon. Herein, we report an asymptomatic large mural thrombus in normal AA which was successfully removed by surgery.

## Case presentation

**Fig. 1:**
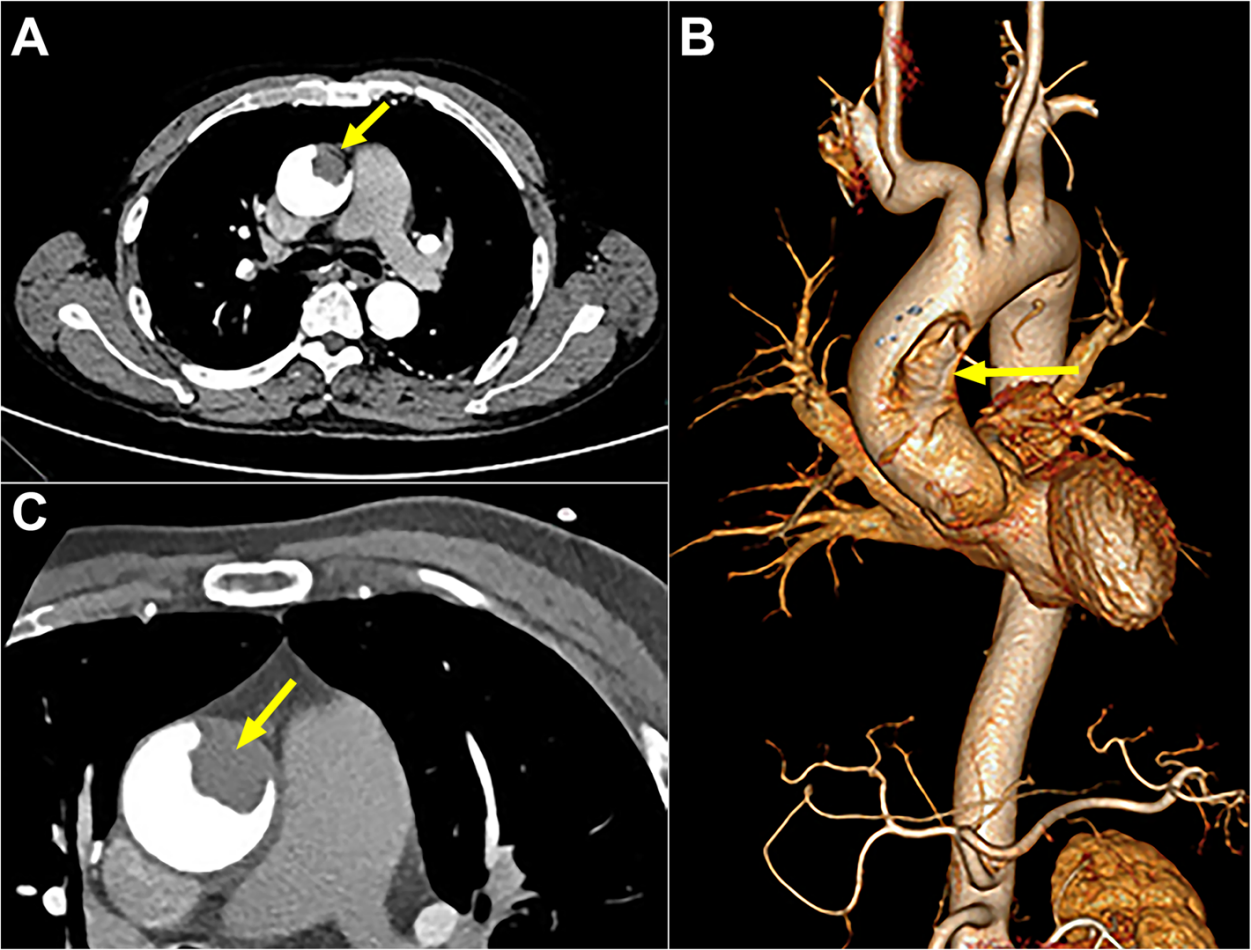
Preoperative imaging. **A** Thoracic contrast-enhanced computed tomography showed a low-density defect(arrow) with no enhancement in the left anterior wall of ascending aorta; **B** The 3-dimensional reconstruction displayed a long oval mass with broad basement(arrow); **C** Coronary computed tomography angiography showed that there was no occlusion of coronary ostia and confirmed a sessile mass with no mobility(arrow)

A 59-year-old man complaining about right lower limb pain and swelling was transferred to our institution for an ascending aortic mass which had been found incidentally in local hospital. Extremity ultrasound confirmed deep vein thrombosis in his right lower limb, which was responsible for his suffering. He had a past history of massive pulmonary embolisms, which were reduced obviously after 5-month course of oral anticoagulant. Except smoking, he had no other risk factors like hypertension, diabetes mellitus and dyslipidemia and he took no medication at that time. The thoracic contrast-enhanced computed tomography(CT) showed a low-density defect with no enhancement in the left anterior wall of AA(Fig. 1A) and the 3-dimensional reconstruction displayed a more detailed spatial location of the mass(Fig. 1B). Apart from this, no evidence of aortic dissection, atherosclerotic change or intramural hematoma had been observed. Coronary computed tomography angiography was performed due to its peculiar location, having the potential to block the coronary ostium. Although no coronary ischemia was been found, it confirmed the presence of the non-floating mass(Fig. 1C). The normal result of positron-emission tomography(PET) and tumor biomarkers reduced the possibility of a malignant tumor. Both the blood clotting tests including fibrinogen, prothrombin time, activated partial thromboplastin time, thrombin time and international normalized ratio and the activity detection of clotting factors II, V, VII ~ XII showed no abnormal findings, while the D-dimer level(4400 μg/L) was much higher than the normal range(80-500 μg/L). Additionally, the nucleic acid test for COVID-19 was negative.

**Fig. 2:**
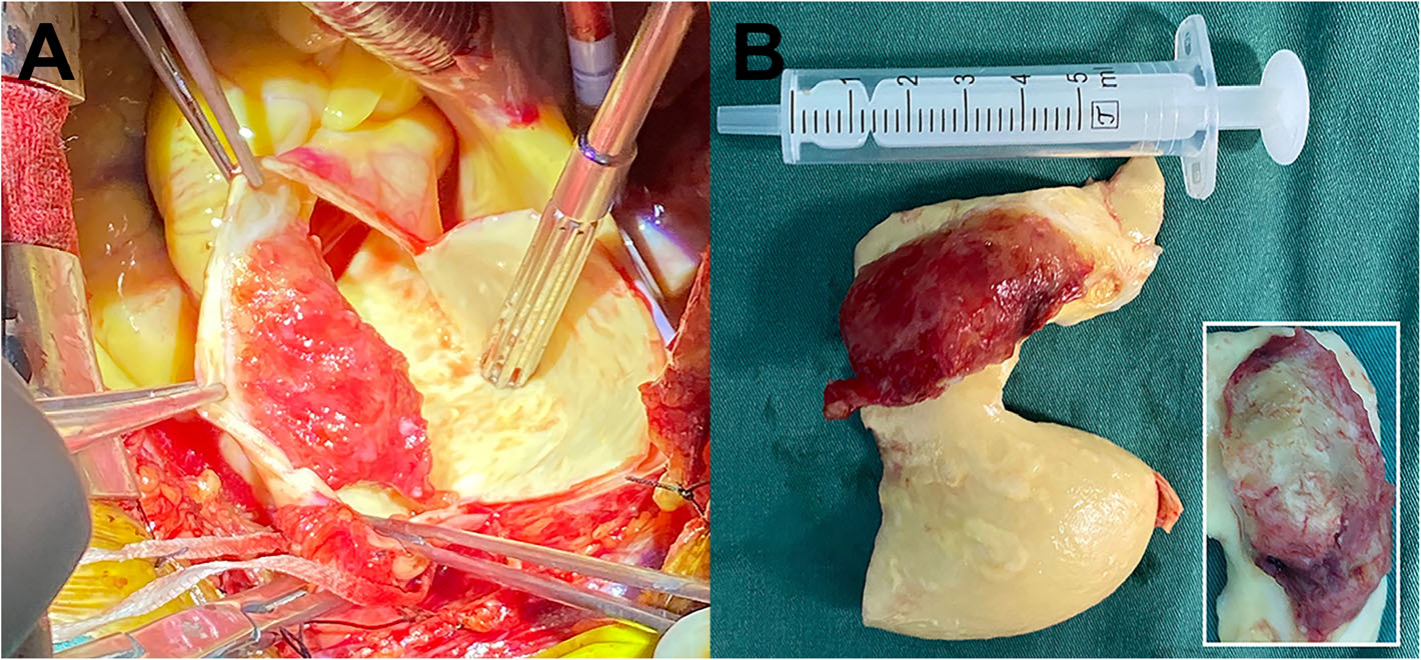
Intraoperative macroscopic findings. **A** A sessile mass was seen attached at the left anterior wall after aortotomy, with a bumpy and jelly-like appearance; **B** The mass measuring approximately 3.0 × 1.5 × 2.2 cm was completely excised with a segment of ascending aorta, gray-yellow intangible substance could be seen after section

Given that the large thrombus in the AA with high risks of fatal thrombotic complications, surgical removal was executed. The surgery was performed through a median sternotomy and then cardiopulmonary bypass was established by cannulation of the right axillary artery and right atrium. After AA cross clamped, it was incised under cardiac arrest and a sessile mass located on the left anterior wall was identified (Fig. 2A). The mass was resected completely with the involved segment of AA, which was replaced with a 26 mm-diameter Dacron graft subsequently. The mass was approximately 3.0 × 1.5 × 2.2 cm in size (Fig. 2B) and gray-yellow substance could be seen after section (lower right in Fig. 2B). There was no atherosclerotic lesion on the excised aortic wall even at the implant site. Histopathological examination confirmed the mass was a white thrombus with intima exfoliation and no evidence of malignancy had been found. He had an uneventful recovery and discharged 14 days after surgery. Heparin and aspirin were applied as anticoagulant therapy during hospitalization, which changed to 3-month course of rivaroxaban when he was discharged.

## Discussion

Mural thrombi in the non-aneurysmal and non-atherosclerotic AA are extremely rare. Although some of those have been reported without significant signs of aortic atherosclerosis on preoperative imaging, they have been found attached at atherosclerotic lesions after aortotomy [1], which were still caused by atherosclerosis essentially. In our case, no atherosclerotic plaque has been observed on the excised aortic wall even at the implant site. The cause of a large mural thrombus forming in high-speed blood flow environment such as AA arouses our great interest. To our best knowledge, the common risk factors include smoking, steroid use such as taking oral contraceptives, and hypercoagulable states like pregnancy and collagen disease [2], among which smoking can even be a sole risk of aortic thrombus [3]. The mechanism of thrombosis in the normal AA remains unclear, but different from those based on atherosclerotic plaque disruption, the thrombus in the normal AA of relatively young(aged<60 years) patients may form on the exfoliated endothelium [2, 4].

As for diagnostic tools, contrast-enhanced CT should be considered as the first choice because it can clearly show whether the aorta has dissection, hematoma or atherosclerosis, and whether the mass displays enhancement. Moreover, the size and location of the mass can be visually seen on reconstructive images. In our case, since the asymptomatic patient was incidentally detected a sessile mass in his non-aneurysmal and non-atherosclerotic AA, the possibility of ascending aortic tumor(e.g. sarcoma) should be taken into account. Thus, we applied PET to exclude the malignancy and evaluate the necessity of surgery. Besides, although transesophageal echocardiography is effective for evaluating the ascending aortic thrombus, this invasive procedure is very uncomfortable for patients and related to possible complications such as gastrointestinal perforation and aspiration pneumonia [5], which is more suitable for detecting the location of thrombus and evaluating its mobility intraoperatively. Magnetic resonance imaging can clearly show the level of the aorta but it takes a long time and seems more suitable for stable patients [1].

At present, there is no corresponding guidelines or consensus on the treatment of the ascending aortic thrombus. There are two main treatment approaches: surgery and conservative therapy with anticoagulant. Endovascular treatment and thrombus aspiration have been also applied in recent years. Anticoagulant should be regarded as the therapeutic cornerstone whether surgery or not, especially appropriate for the patients with high risks of surgical intervention. Some researchers have reported good outcomes of anticoagulant therapy alone [6]. However, it may result in fatal embolism when the thrombus is larger than 1 cm [7]. As far as we concerned, surgery is supposed to be a preferred choice when the thrombus is large and floating, or occurrence of systemic embolism due to conservative treatment failure. The types of surgery mainly include simple thrombus resection and aorta replacement. Simple resection is often used when a pedunculated thrombus attached at aortic wall with a narrow stalk [8] while a large sessile thrombus is apt to be dealt with the involved segment of aorta replacement to avoid recurrence [9]. Considering his previous thrombotic events, we thought he may have high risk of distal embolism during conservative treatment. And we could not exclude the possibility of tumor completely so we performed surgical resection plus replacement. Of note, circulatory arrest should be achieved to avoid cross clamping the AA if the location of the thrombus is superior. Heparin and aspirin were given postoperatively, which changed to rivaroxaban after discharge. Endovascular treatment and thrombus aspiration may be considered as an alternative to patients unable to tolerate surgery. However, the efficacy and safety of these new techniques need more clinical experience to be evaluated since any endovascular manipulation has risk of injuring aortic wall or unstable thrombus shedding further causing distal embolism.

## Conclusions

We presented a large and sessile mural thrombus in the normal AA of an asymptomatic patient. Ascending aortic thrombus should be taken into consideration when meet with a relatively young patient showing clinical manifestations of arterial embolism. Although anticoagulant therapy is generally recommended, we advocate that surgery should be performed aggressively when the thrombus is large or floating to avoid severe embolic complications or recurrence.

## Data Availability

Please contact author for data requests.

## Abbreviations

AA: ascending aorta
CT: computed tomography
PET: positron-emission tomography
